# Effect of samul-tang on female fertility via RAS signaling pathway in ovaries of aged mice

**DOI:** 10.18632/aging.203150

**Published:** 2021-06-06

**Authors:** Jihyun Kim, Sooseong You

**Affiliations:** 1 Clinical Medicine Division, Korea Institute of Oriental Medicine, Daejeon, Republic of Korea

**Keywords:** aging, samul-tang, ovarian reserve, pregnancy, mice

## Abstract

Samul-tang (SM), a traditional herbal medicine, is used to treat age-related human conditions, such as infertility and menstrual irregularities. The mechanism underlying the role of SM in ovary function needs elucidation. In this study, the influence of SM administration on the ovarian reserve of aged mice was investigated. Female BALB/c mice (8 and 40 weeks-old) were administered with distilled water (young or old group) or SM for 4 weeks. SM administration prevented age-related ovarian follicle loss in mice. Quality of oocytes and blastocysts were enhanced in SM-administrated mice compared to those of non-treated old mice. Further, SM administration increased the pregnancy rate and number of litters. SM triggered changes in aging-related genes that are linked to the RAS-mediated pathway. Thus, we demonstrate that SM can be used to increase the oocyte yield in aged women, potentially improving age-related cognitive decline in the ovarian reserve.

## INTRODUCTION

Aging remains associated with a variety of diseases, including infertility, degenerative diseases, and cancer [1]. Female ovarian reserve has been reported to decline progressively with advancing age [2]. Women in the elderly age group have a decreased follicle pool of less than 1,000 and show irregular ovulatory cyclic changes, the first clinical signs of ovarian aging [3, 4]. Ovarian aging results in a poor response to ovarian stimulation leading to reduced number and quality of remaining oocytes in women [5, 6]. In particular, older women (> 40 years), are subjected to increased risks of chromosomal abnormalities in oocytes and adverse pregnancy outcomes [7, 8]. The most commonly faced challenge has been the effective management and improvement of age-related decline in female reproductive potential [9].

Recent research has focused on developing effective fertility preservation strategies, such as ovarian tissue and oocyte cryopreservation [10]. However, such attempts are likely to be effective only in women younger than 35 years and with good ovarian reserve. Because older women with poor ovarian responses (POR) have a declining oocyte yield in *in vitro* fertilization, high-dose follicle-stimulating hormone (FSH) protocols are unsuccessful [11]. Thus, differential pharmacological strategies are required for women to improve the ovarian reserve.

Alternative options in integrative medicine treatment using natural herbs are considered effective for elderly women with POR. Herbal combinations have been used to manage infertility symptoms in human and rodent models [12–14]. Samul-tang (SM) is a well-known mixed herbal medicine comprising equal proportions of *Paeonia lactiflora*, *Liqusticum striatum*, *Rehmannia glutinosa*, and *Angelica gigas*. Pharmacological effects of SM include anti-inflammatory, antioxidative, anti-stress, and anti-cancer effects [15]. The herb is commonly prescribed to women with gynecological disorders, such as irregular menstruation and postmenopausal syndrome, often clinically observed in older women with POR [16]. Anti-aging effects of three compounds, including *P. lactiflora*, *R. glutinosa*, and *A. gigas*, have been reported using human cells and animal models [17–19]. Although herbal medicines are traditionally known to improve fertility-related parameters, scientific evidence supporting their use for age-related infertility is limited.

In this study, the influence of SM administration on ovarian reserve for four weeks in old-aged mice was investigated. Further, the compensatory mechanisms of SM in age-related functional decline in ovary were uncovered using transcriptomic analysis.

## RESULTS

### SM improved age-related decline of serum AMH and FSH level

**Figure 1:**
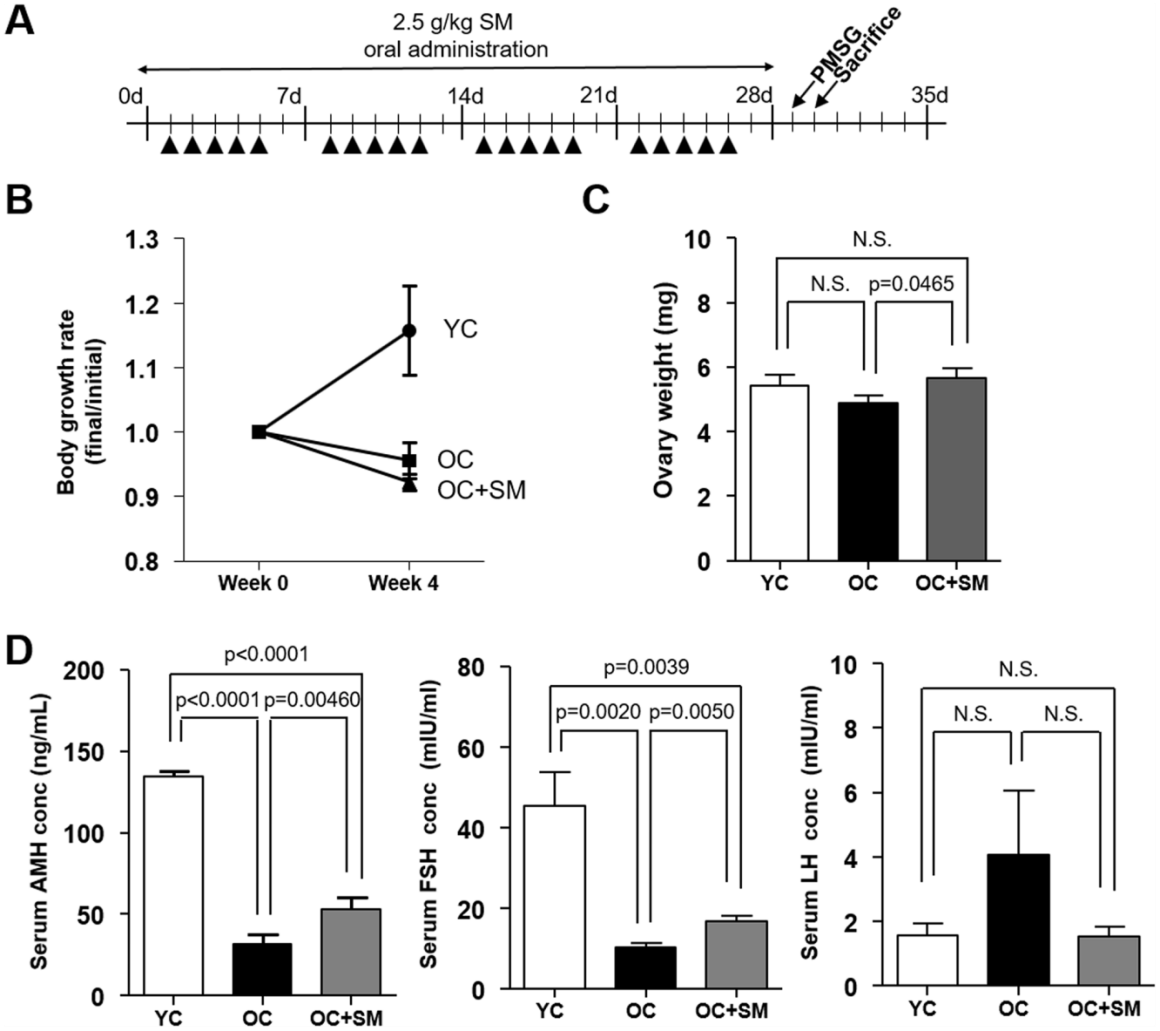
**Body weight changes and serum hormone levels in mice after Samul-tang (SM) administration.** (**A**) Eight-week-old mice were orally administered distilled water (n=6, YC group). Forty-week-old mice were orally administered distilled water (n=6, OC group) or 2.5 g/kg of SM (n= 7, OC+SM group) five times a week for four weeks. Post SM administration, the mice were weighed and hormonal assessment was performed. (**B**) Body weight changes. (**C**) Ovary weight. (**D**) Serum levels of anti-Müllerian hormone (AMH), follicle-stimulating hormone (FSH), and luteinizing hormone(LH). Data are presented as mean ± standard error of the mean. Statistical analysis was performed using the Student’s *t*-test.

Body weight changes in mice of each group were measured for four weeks since SM administration (Figure 1A). SM had no effect on the body weight (Figure 1B). However, decreased ovary weight associated with aging was regained by SM administration for four weeks (Figure 1C).

Next, we measured the serum levels of anti-Müllerian hormone (AMH), FSH, and luteinizing hormone (LH) in each mouse group. OC mice (40-week-old mice orally administered distilled water) exhibited low AMH levels resulting in a decreased ovarian reserve (Figure 1D). SM administration in OC+SM mice significantly enhanced the serum AMH levels. Although serum LH levels did not change, FSH levels increased significantly in OC+SM mice compared to those in OC mice (Figure 1D). It is likely that the production of more FSH directly stimulates the granulosa cells for follicular growth and development [20].

### SM prevented aged-related ovarian follicle loss

**Figure 2:**
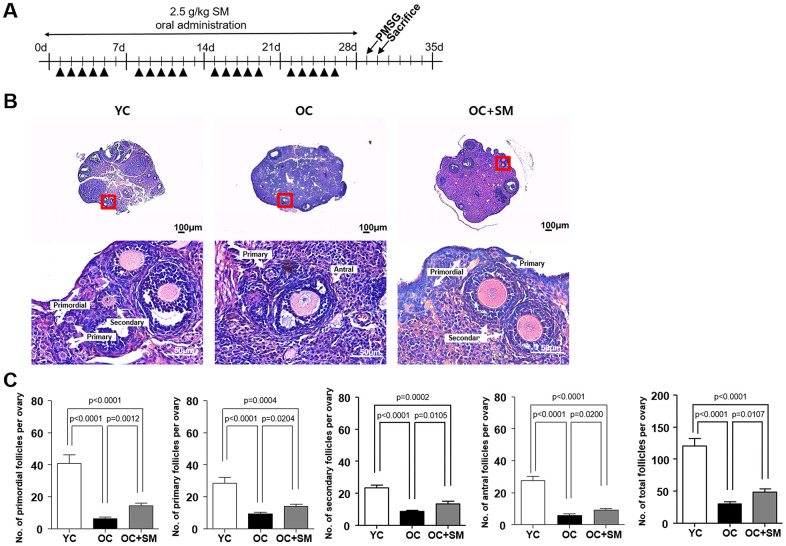
**Histological analysis of ovarian follicles in mice after Samul-tang (SM) administration.** (**A**) Eight-week-old mice were orally administered distilled water (n = 6, YC group). Forty-week-old mice were orally administered distilled water (n = 6, OC group) or 2.5 g/kg of SM (n = 7, OC+SM group) five times a week for four weeks. (**B**) Post SM administration, both mouse ovaries were assessed histologically. (**C**) Number of ovarian follicles in different stages and the total number of ovarian follicles. Data are presented as mean ± standard error of the mean. Statistical analysis was performed using the Student’s *t*-test.

To investigate the protective effects of SM on ovarian follicle growth under aging conditions, a histological analysis of the ovaries excised from the mice after SM administration was performed (Figure 2A). Histological changes in YC (8-week-old mice orally administered distilled water), OC, and OC+SM mice were observed in ovarian tissues (Figure 2B). Compared to the YC mice, aged mice showed an overall reduction in ovarian follicles. OC+SM mice showed higher number of follicles at all stages than the OC mice (Figure 2C). These results suggest that SM administration prevents depletion of the primordial follicle pool.

### SM ameliorated aged-related impairment of oocyte quality

**Figure 3:**
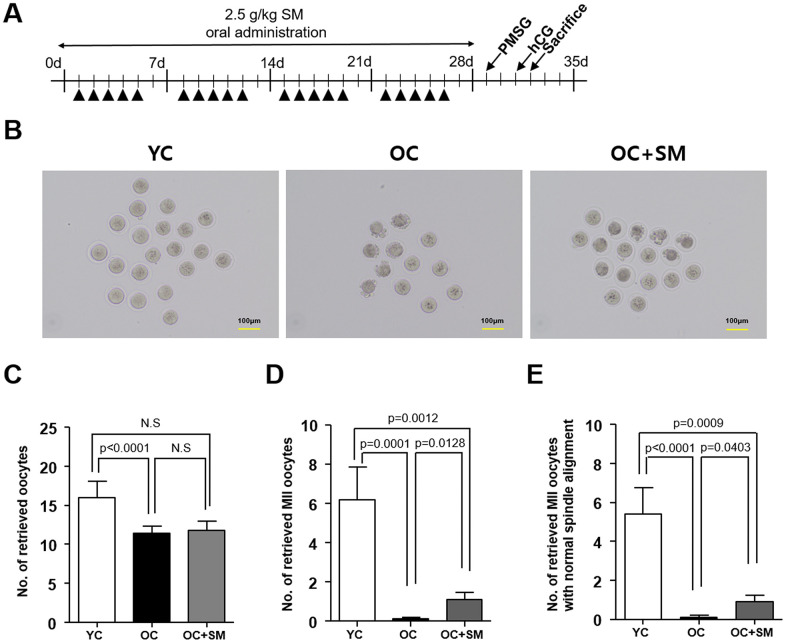
**Quality and quantity of mouse oocytes retrieved after Samul-tang (SM) administration.** (**A**) Eight-week-old mice were orally administered distilled water (n = 6, YC group). Forty-week-old mice were orally administered distilled water (n = 10, OC group) or 2.5 g/kg of SM (n = 10, OC+SM group) five times a week for four weeks. Post SM administration, the mice were superovulated via hormonal stimulation. (**B**) Oocytes retrieved from the YC, OC, and OC+SM mice at 18 h after hCG injection. Number of retrieved oocytes (**C**), mature metaphase II (MII) oocytes (**D**), and MII oocytes with normal chromosomal and spindle alignment (**E**), retrieved from the three different groups of mice. Data are presented as mean ± standard error of the mean. Statistical analysis was performed using the Student’s *t*-test.

To investigate the protective effects of SM on oogenesis under aging conditions, mice were hormonally superovulated to collect the oocytes (Figure 3A). Thereafter, the quantity and quality of oocytes were assessed (Figure 3B). Number of total oocytes retrieved from the aged mice was significantly lower than that retrieved from the YC mice (*P*< 0.05; Figure 3C). However, the number of mature metaphase II (MII) oocytes, with normal chromosomes and well-organized spindle alignments, was significantly higher in OC+SM mice than in OC mice (Figure 3D, 3E). These results indicate that SM administration could improve the quality of oocytes in aged mice.

### SM reversed aged-induced changes in mRNA expression in mouse ovaries

**Figure 4:**
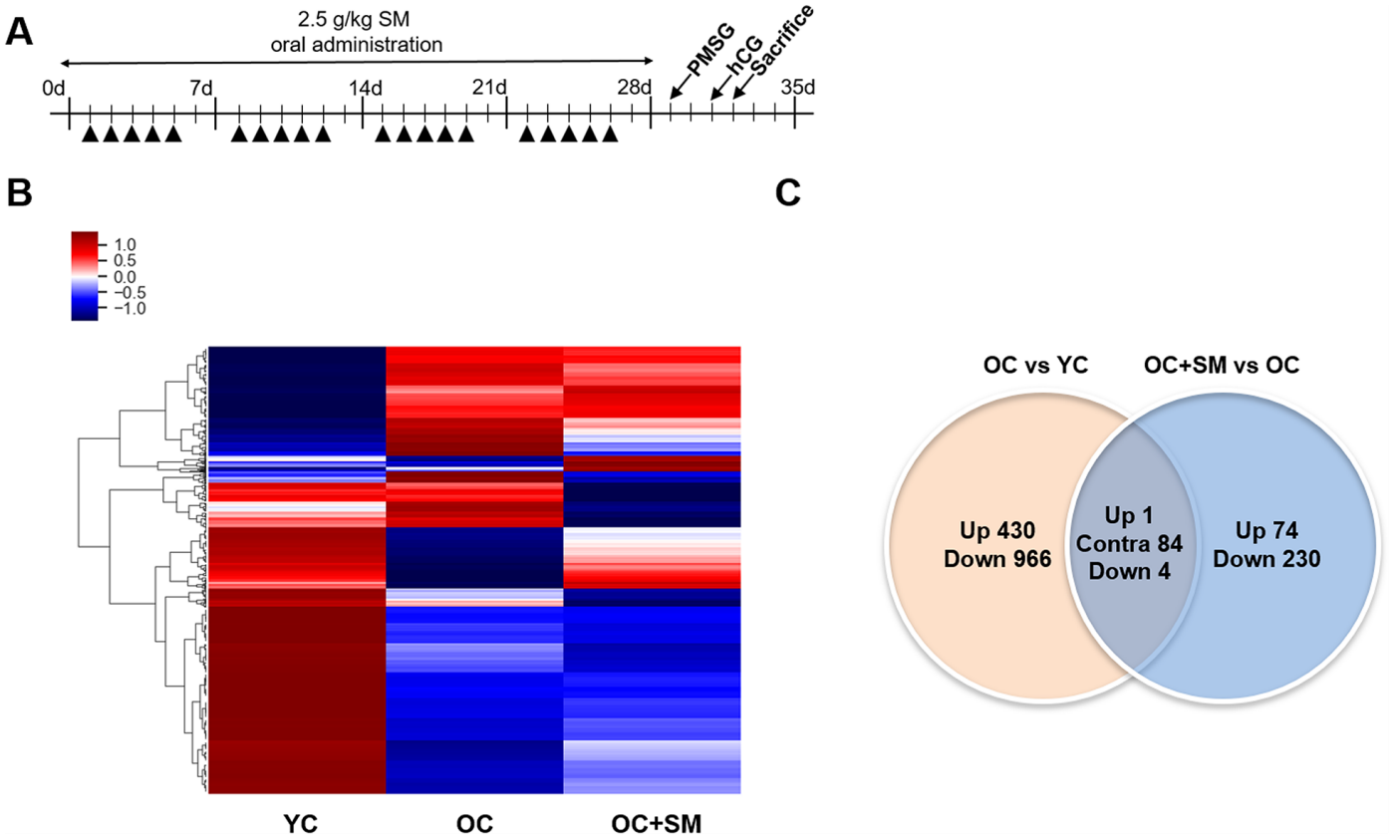
**Hierarchical clustering and analysis of differentially expressed messenger RNAs (mRNAs).** (**A**) QuantSeq 3’ mRNA analysis was performed to compare the gene expression in ovulated ovaries of YC (n = 6), OC (n = 6), and OC+SM (n = 6) mice. (**B**) Hierarchical clustering among the mRNA expression profiles showing 2,389 differentially expressed mRNAs in the three groups, with a fold-change >1.5 and *P*< 0.05. (**C**) Venn diagram presenting the numbers of differentially expressed mRNAs between OC vs. YC and OC+SM vs. OC pairs. YC: 8-week-old control mice; OC: 40-week-old mice; OC+SM: 40-week-old mice orally administered Samul-tang. Up, upregulated genes between compared sets; Contra, contraregulated genes between compared sets; Down, downregulated genes between compared sets.

**Figure 5:**
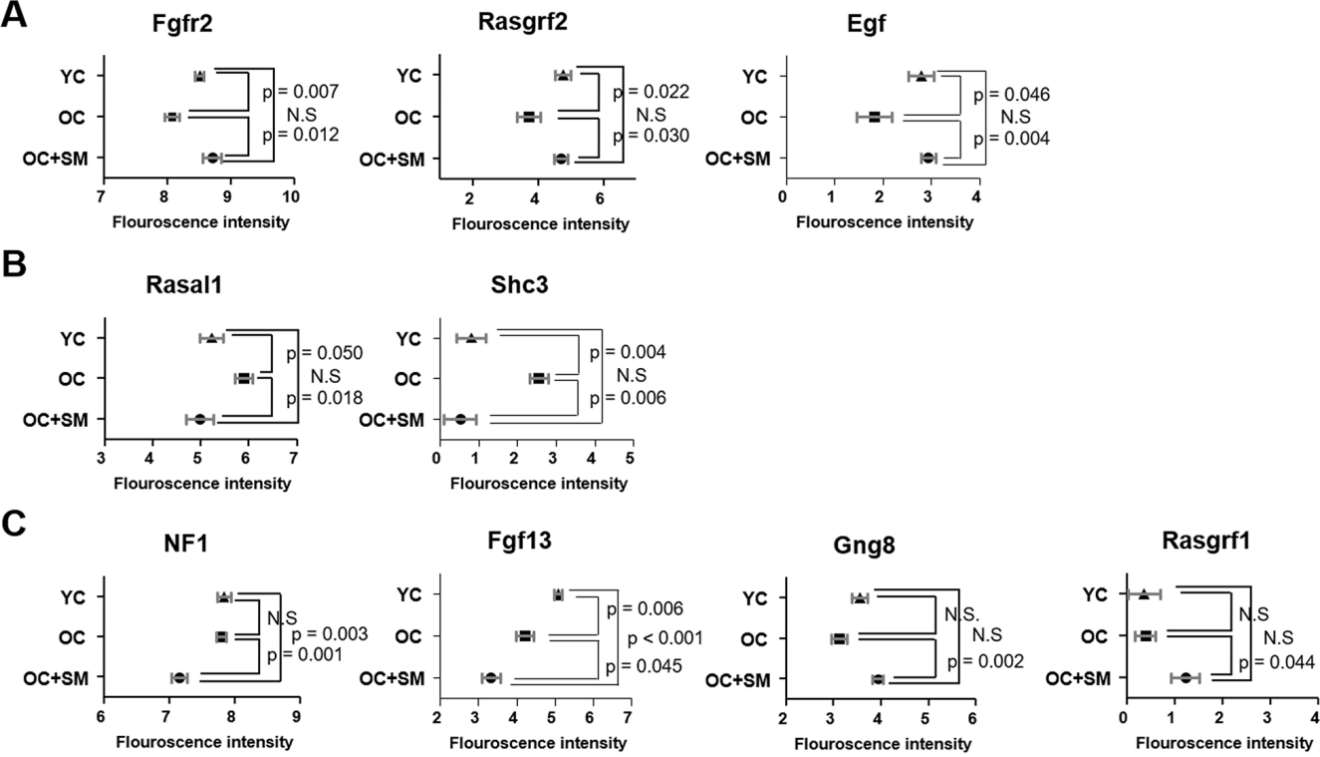
**Differentially expressed mRNAs involved in RAS signaling pathway.** Fluorescence intensities indicating the expression levels of *Fgfr2, Rasgrf1, Egf, Rasal1, Sch3, Nf1, Fgf13, Gng8,* and *Rasgrf2* in the ovaries of YC, OC, and OC+SM mice. (**A**) Aging-induced downregulated expression of *Fgfr2, Rasgrf2* and *Egf* were restored with SM administration. (**B**) Aging-induced upregulated expression of *Rasal1* and *Sch3* were restored with SM administration. (**C**) Expression of *Nf1, Fgf13, Gng8,* and *Rasgrf1* were changed by SM administration in OC mice. Data are presented as mean ± standard error of the mean. Statistical analysis was performed using the Student’s *t-*test. YC: 8-week-old control mice; OC: 40-week-old mice; OC+SM: 40-week-old mice orally administered Samul-tang.

**Figure 6:**
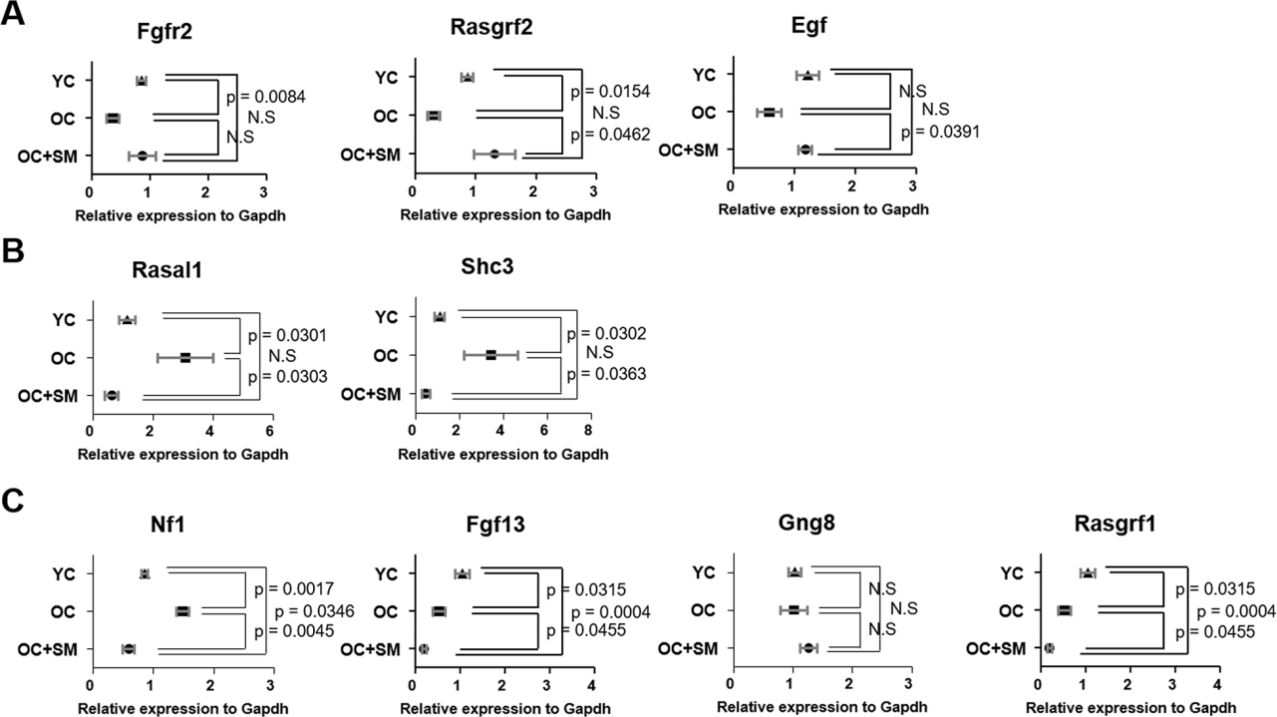
**Validation of expression of differentially expressed mRNAs involved in ovarian function.** Quantitative polymerase chain reaction was performed to validate the expression of *Fgfr2, Rasgrf1, Egf, Rasal1, Sch3, Nf1, Fgf13, Gng8,* and *Rasgrf2* in the ovaries of YC, OC and OC+SM mice. (**A**) Aging-induced downregulated expression of *Fgfr2, Rasgrf2* and *Egf* were restored with SM administration. (**B**) Aging-induced upregulated expression of *Rasal1* and *Sch3* were restored with SM administration. (**C**) Expression of *Nf1, Fgf13, Gng8,* and *Rasgrf1* were changed by SM administration in OC mice. Data are presented as mean ± standard error of the mean. Statistical analysis was performed using the Student’s *t-*test. YC: 8-week-old control mice; OC: 40-week-old mice; OC+SM: 40-week-old mice orally administered Samul-tang.

QuantSeq 3’ messenger RNA (mRNA) sequencing was performed to compare the mRNA expression patterns in ovulated ovaries obtained from YC, OC, and OC+SM mice (Figure 4A). Hierarchical clustering analysis revealed 2,389 marked differentially expressed genes (DEGs) in the three mice groups, with fold changes >1.5 (*P*< 0.05; Figure 4B). We specifically focused on 393 DEGs in the OC+SM vs. OC group (Figure 4C; blue circle). Bioinformatic analysis was performed for 122 upregulated genes (31.0%) and 271 downregulated genes (69.0%) (Supplementary Tables 1, 2). Kyoto Encyclopedia of Genes and Genomes pathway analysis of OC and OC+SM mice datasets revealed that the DEGs were involved in the RAS signaling pathway (Table 1). Expression of nine genes associated with the RAS signaling pathway was analyzed. The genes include fibroblast growth factor receptor 2 (*Fgfr2*), guanine nucleotide binding protein gamma 8 (*Gng8*)*,* RAS protein activator like 1 (*Rasal1*), RAS protein-specific guanine nucleotide-releasing factor 1 and 2 (*Rasgrf1* and *Rasgrf2*)*,* neurofibromatosis 1 (*Nf1*), fibroblast growth factor 13 (*Fgf13*), Src homology 2 domain-containing transforming protein C3 (*Shc3*), and epidermal growth factor (*Egf*). *Fgfr2*, *Rasgrf2*, and *Egf* were downregulated in OC mice; however, their expression levels were restored with SM administration (Figure 5A). Likewise, SM administration also restored the expression levels of *Shc3* and *Rasal1*, which were initially upregulated in OC mice (Figure 5B). Expression of *Nf1* and *Fgf13* in the ovaries of OC+SM mice decreased significantly compared to that in the ovaries of YC and OC mice (Figure 5C). *Gng8* and *Rasgrf1* expressions increased significantly in OC+SM mice than in the ovaries of OC mice (Figure 5C). These genes were validated by quantitative polymerase chain reaction (qPCR) using the fluorescent probe-based TaqMan assay (Figure 6). Different expression patterns of *Gng8* and *Rasgrf1* were observed in sequencing data. Given the filtering of transcriptomics technologies for quality measures, including fold-change >1.5 and *P* < 0.05, this study obtained varying results from sequencing and qPCR analysis [21]. These findings suggest that SM administration ameliorates the age-related deterioration of ovarian function via genetic regulation.

**Table 1:** Functional annotation of differentially expressed genes.

**Term**	**P-value**	**Genes**
**Mmu05034: Alcoholism**	5.58E-04	*Gng8, Hist4h4, Hist1h4a, Npy, Hist1h2bf, Hist2h2ac, Creb3l2, Hist1h4d, shc3, Hist1h2bq*
**Mmu04014: Ras signaling pathway**	0.00051931	*Fgfr2, Gng8, Rasal1, Rasgrf1, Rasgrf2, Nf1, Fgf13, Shc3, Egf*
**Mmu04911: Insulin secretion**	0.0179786	*Gcg, Slc2a2, Creb3l2, Cacna1f, Pclo*
**Mmu05322: Systemic lupus erythematosus**	0.0286335	*Hist4h4, Hist1h4a, Hist1h2bf, Hist2h2ac, Hist1h4d, Hist1h2bq*

Kyoto Encyclopedia of Genes and Genomes analysis was performed to identify the potential signaling pathway of the differentially expressed genes.

### SM improved aged-induced decline in blastocyst developmental competence

**Figure 7:**
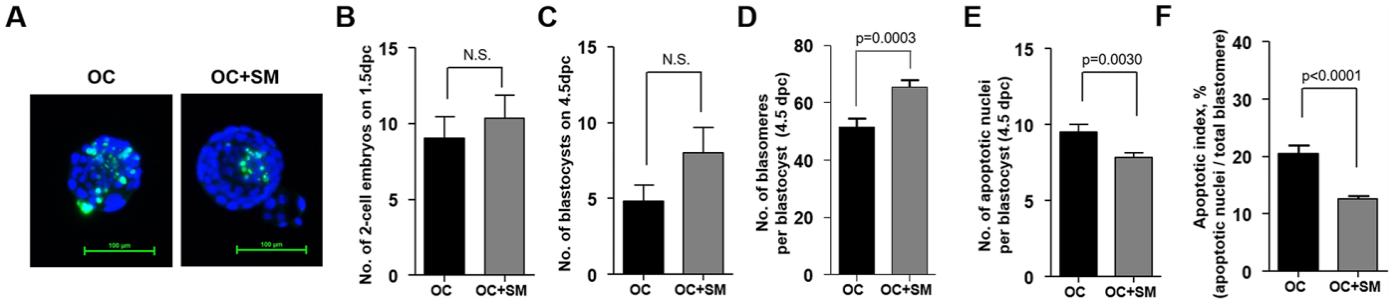
**Quality and quantity of mouse blastocysts retrieved after Samul-tang (SM) administration.** Forty-week-old mice were orally administered distilled water (n = 13, OC group) or 2.5 g/kg of SM (n =13, OC+SM group) five times a week for four weeks. Post SM administration, the mice were superovulated via hormonal stimulation and mated with fertile males. At 1.5 days post coitum (dpc), 2-cell embryos were collected and cultured up to blastocyst stage for three days. (**A**) Blastocyst quality was evaluated by assessing the stained nuclei and DNA fragmentation in blastocysts from each mice group. Number of collected 2-cell embryos at 1.5 dpc (**B**) and cultured blastocysts at 4.5 dpc (**C**). (**D**–**F**) Blastocyst developmental competence was assessed from the number of blastomeres and apoptotic nuclei, and apoptotic index. Data are presented as mean ± standard error of the mean. Statistical analysis was performed using the Student’s *t*-test.

To investigate whether SM enhances the developmental competence under aging conditions, superovulated mice were mated with fertile males. At 1.5 days post coitum (dpc), 2-cell embryos were flushed from the oviduct and cultured to obtain blastocysts for three days. At 4.5 dpc, blastomeres and DNA fragmentation in blastocysts were stained to assess developmental competence (Figure 7A).

No significant difference in the number of 2-cell embryos and developed blastocysts was observed between 1.5 to 4.5 dpc (Figure 7B, 7C). However, the number of blastomeres per blastocyst increased significantly. Number of apoptotic cells was lower in OC+SM mice than in OC mice (Figure 7D, 7E). Apoptotic index decreased in blastocysts obtained from OC+SM mice compared to OC mice (Figure 7F). These results indicate that blastocysts with elevated total cell number and decreased apoptotic index could affect the implantation potential *in vivo* and pregnancy outcomes.

### SM improved implantation potential in aged mice

**Figure 8:**
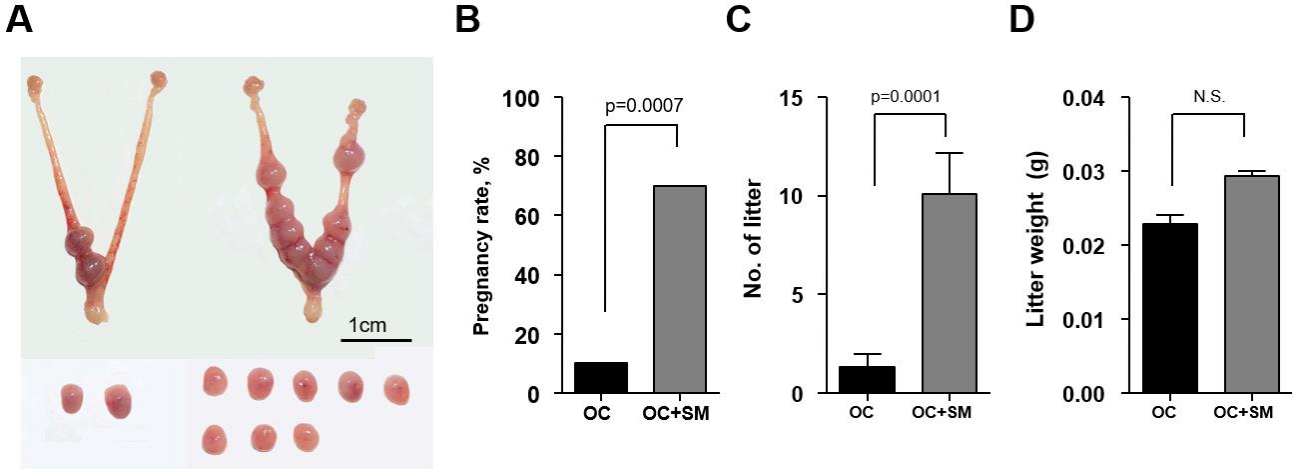
***In vivo* implantation potential after Samul-tang (SM) administration.** Forty-week-old mice were orally administered distilled water (n = 20, OC group) or 2.5 g/kg of SM (n = 10, OC+SM group) five times a week for four weeks. Post SM administration, the mice were superovulated via hormonal stimulation and mated with fertile males. (**A**) At 9.5 days post coitum (dpc), the uterus from mice of both the groups was collected to assess the implantation potential. (**B**) Pregnancy rates. (**C**) Number of litters. (**D**) Weight of litters. Data are presented as mean ± standard error of the mean. Statistical analysis was performed using the Student’s *t*-test. The significance of difference in pregnancy rates was determined using Fisher’s exact test.

To investigate whether SM enhances the implantation potential *in vivo* under aging conditions, superovulated mice were mated with fertile males. Pregnancy rate, number and weight of litter were assessed at 9.5 dpc (Figure 8A).

The pregnancy rate was significantly higher in OC+SM mice than in OC mice (Figure 8B). Furthermore, SM administration enhanced the number of litters having weight similar to OC mice weight (Figure 8C, 8D).

## DISCUSSION

Age-related fertility is characterized by gradual decline in ovarian follicle quantity and oocyte quality since birth [22]. Decrease in fertility enhances the risk of reproductive failure [23]. Pharmacological research on anti-ovarian aging agents for women who suffer from POR or premature ovarian failure has gained significant momentum [24]. Thus, new strategies or approaches to prevent ovarian aging are urgently needed.

Progressive decline in tissue homeostasis and biological function with aging contributes to increased risk of miscarriage, degenerative diseases, and death [25]. Some of the well-known causes of aging include oxidative damage caused by generation of free radicals and reactive oxygen species (ROS), and genetic instability-driven genome damage [26]. Oxidative deterioration of DNA, proteins, and lipids by ROS and free radical overproduction has been reported in aged ovaries [27]. Genetic defects in DNA repair systems are considered responsible for the senescent phenotype causing aging syndromes, such as premature aging [28, 29]. SM comprises numerous active components, including gallic acid, paeoniflorin, and ferulic acid. These compounds contain several hydroxyl groups that scavenge the overproduced free radicals [30, 31].

RAS signaling modulates the balance between ROS and antioxidants to prevent cellular senescence or apoptosis [32, 33]. Balanced GDP/GTP cycling maintains the RAS-mediated signaling, which plays a critical role in controlling the normal cellular proliferation and survival. RAS also plays a critical role in the events associated with ovulation and luteinization in preovulatory follicles [34]. Its activity is regulated by multiple guanine nucleotide exchange factors, such as RAS-GRF2 and GTPase-activating proteins, such as Nf1 and Rasal1 [35]. The qPCR results revealed that downregulated *Rasgrf2* and upregulated *Rasal1* and *Shc3* inactivate the RAS-mediated signaling cascades in ovarian follicle development and oocyte maturation in aged ovaries. Interestingly, the altered expression of these genes was restored with SM administration in aged mouse ovaries. EGF also acts through the RAS/ERK pathway to regulate protein homeostasis by promoting the expression of antioxidant genes [36]. EGF signaling is not only essential for normal ovarian steroid oogenesis and oocyte maturation but is also required to induce cumulus cell expansion *in vitro* during the ovulatory process [37]. *Egf* expression in aged ovaries receiving SM administration was significantly upregulated compared to that in untreated ovaries. SM likely prevents cell senescence and enhances ovarian reserve by activating Ras in aged women.

Active RAS-mitogen-activated protein kinase pathway can largely regulate FGF signaling, which is known to be associated with several developmental processes, including cellular proliferation, differentiation, angiogenesis, and migration [38]. *Fgf13*, one of the genes whose expression is decreased by SM, plays an important role in the reconstruction and degradation of the extracellular matrix in infertile aged women [39]. In addition, bidirectional dialog between Fgf2 in oocytes and several Fgfr in granulosa cells is essential for early ovarian folliculogenesis and granulosa cell growth [40]. Restored *Fgfr2* expression by SM could pose as an evidence for potential oocyte developmental competence. Functional assays revealed that loss of endothelial Fgfr1 and Fgfr2 results in impaired neovascularization in adult mice [41]. Aged patients exhibit decreased ovarian stromal blood flow owing to a marked reduction in the number and caliber of blood vessels with changes in endothelial cells [42]. Insufficient supply of growth factors owing to decreased blood flow causes follicular arrest and ovarian fibrosis in aged women with POR [43, 44]. SM-induced changes in FGF signaling could drive tissue repair in aged ovaries pre- or post-ovulation [45, 46].

Interestingly, the pregnancy rates were higher in aged mice receiving SM administration. Successful implantation requires good quality of implanting embryo and appropriate structural and functional remodeling of the endometrium [47]. Although SM administration did not increase the number of 2-cell embryos and blastocysts, it enhanced the scope to implant competent blastocysts with more cell divisions.

This study aimed to prevent age-specific decline in functional ovarian reserve using an aged mouse model. However, functional analysis and clinical studies are still warranted to develop strategies or approaches that could improve conditions or delay ovarian aging. Identification of the potential regulatory genes highlighted the SM-induced epigenetic regulation in age-related cognitive decline in ovarian reserve and implantation potential. SM restores the homeostatic balance to revitalize ovarian function under aging condition through the RAS signaling pathway. In conclusion, SM could be helpful in increasing the oocyte yield in aged women by potentially improving the age-related cognitive decline in ovarian reserve.

## MATERIALS AND METHODS

### Mice

All experiments and analyses were conducted in accordance with the relevant guidelines and regulations. Experimental protocols concerning animals were approved by the Institutional Animal Care and Use Committee of the Korea Institute of Oriental Medicine, Daejeon, Korea (approval number 19-061). Female BALB/c mice aged 8 and 40 weeks (Central Lab Animal Inc., Seoul, Korea) were housed under specific pathogen-free conditions. To investigate the effect of SM (Hanpoong, Iksan, Korea), the old mice were orally administered either distilled water (OC group) or 2.5 g/kg of SM (OC+SM group) five times a week for four weeks.

Mice from the OC and OC+SM groups were separated within the same cage to synchronize their hormonal cycles [48]. All mice were sacrificed after completion of SM administration. Ovaries were removed, weighed, and immediately placed in 4% paraformaldehyde (Biosesang, Seongnam, Korea) or liquid nitrogen for histological or RNA sequencing, respectively.

### Enzyme-linked immunosorbent assay (ELISA) for hormonal assessment

Post SM administration, blood samples were collected from the mice and sera were separated and stored at -80 ° C until analysis. The serum concentrations of AMH, FSH, and LH were measured using hormone-specific ELISA kits from Ansh Lab (Webster, TX, USA), Cusabio Biotech Co. (Wuhan, China), and Endocrine technologies (Newark, CA, USA), respectively, according to the standard protocols and manufacturers’ instructions. For AMH, the inter-assay coefficients of variation (CV) was <10%, with a sensitivity of 0.06 ng/mL. For FSH, both intra- and inter-assay CVs were <15% with a sensitivity of 2.5 mIU/mL. For LH, the intra- and inter-assay CVs were 7% and 15%, respectively, and the functional sensitivity was 5.2 mIU/mL.

### Histological assessment of ovarian follicles

Post SM administration, ovaries from YC, OC and OC+SM groups were serially sectioned to obtain 5-μm-thick tissue sections. These sections were then subjected to hematoxylin and eosin staining. Primordial, primary, secondary, and preovulatory follicles, with visible oocytes, were counted in every tenth stained section to avoid repeated counting of the same follicle. The follicular stages were classified as previously described [49]: primordial follicles, with a single flat layer of granulosa cells surrounding the oocyte; primary follicles, with a single cuboidal granulosa cell layer; secondary follicles, with at least two granulosa cell layers and a theca layer; and preovulatory follicles, with a complete antrum and theca layer.

### Assessment of oocyte quantity and quality

Post SM administration, the mice were superovulated with an intraperitoneal injection of 5 IU of pregnant mare serum gonadotropin (PMSG; Prospec, Rehovot, Israel). Another injection of 5 IU of human chorionic gonadotropin (hCG; Prospec) was administered 48 h later. Oocytes were collected 18 h post-hCG injection in preincubated human tubal fluid medium (Irvine Scientific, CA, USA). Oocytes were fixed with 4% paraformaldehyde (Biosesang), permeabilized with 0.5% Triton X-100 (Sigma–Aldrich, St. Louis, MO, USA) for 10 min, and blocked with phosphate-buffered saline containing 3% bovine serum albumin (GenDEPOT, TX, USA). Thereafter, the oocytes were incubated with a rabbit anti-α-tubulin antibody (1:200; Cell Signaling Technology, MA, USA) and subsequently mounted on slides using VECTASHIELD antifade mounting medium with 4,6-diamidino-2-phenylindole (Vector Laboratories, Peterborough, UK) to visualize the chromosomes using a fluorescence microscope (BX51; Olympus, Tokyo, Japan). Oocytes with well-organized bipolar spindles and tightly aligned chromosomes at metaphase were scored as normal.

### RNA sequencing for mRNA expression

Post SM administration, ovaries were collected from the mice post-ovulation, and total RNA was extracted using TRIzol reagent (Invitrogen, Carlsbad, CA, USA), according to the manufacturers’ protocol. The purity and integrity of the extracted RNA were evaluated using a NanoDrop ND-2000 spectrophotometer (Thermo Fisher Scientific, Waltham, MA, USA) and Agilent 2100 bioanalyzer (Agilent Technologies, Amstelveen, The Netherlands). All samples showed high purity (optical density (OD)_260_/OD_280_> 1.80) and integrity (RNA integrity number >7.0). Sequencing was performed using the Illumina NextSeq 500 platform. A fold-change value >1.5 and a *P-*value <0.05 were considered thresholds to identify DEGs.

### Validation of selected DEGs in the ovaries

To confirm the mRNA microarray results, validation was performed on significant genes of interest (*Fgfr2*, *Gng8, Rasal1, Rasgrf1, Rasgrf2, NF1, Fgf13, Shc3,* and *Egf*) using real-time qPCR. Complementary DNA (cDNA) was synthesized from the extracted total RNA using iScript cDNA Synthesis kit (Bio-Rad Laboratories, Hercules, CA, USA), according to the manufacturers’ protocol. qPCR was performed in a final reaction volume of 20 μL using a QuantStudio 6 Flex Real-time PCR system with fluorescent probe-based TaqMan assays, according to the manufacturers’ protocol (Thermo Fisher Scientific). The cycle threshold was normalized and compared using glyceraldehyde 3-phosphate dehydrogenase as the internal standard.

### Assessment of *in vitro* developmental competence

Post SM administration, the mice were superovulated with an intraperitoneal injection of 5 IU of PMSG, followed by 5 IU of hCG 48 h later. Superovulated mice were mated with fertile male mice, and the day of vaginal plugging was designated as 0.5 dpc. At 1.5 dpc, 2-cell embryos were collected and cultured up to the blastocyst stage for three days. DNA fragmentation was determined by transferase dUTP nick end labeling (Promega, WI, USA). Blastocyst quality was assessed using the following three parameters: number of blastomeres, DNA fragmentation, and apoptotic index per blastocyst. The apoptotic index was calculated for each blastocyst as follows: apoptotic index= (number of TUNEL-positive nuclei/total number of nuclei) × 100.

### Assessment of *in vivo* implantation potential

Post SM administration, superovulation and mating were achieved as described above. The day of vaginal plugging was designated as 0.5 dpc. At 9.5 dpc, pregnant mice were sacrificed to assess the implantation potential following three parameters: pregnancy rate, total number of litter, and litter weight.

### Statistical analysis

Data are presented as mean ± standard error of the mean. The significance of difference between two groups was determined by a Student’s *t*-test using GraphPad Prism version 8.4.0 (GraphPad Software, La Jolla, CA, USA). The significance of difference between the expected frequencies was determined using Fisher’s exact test. A *P*-value < 0.05 was considered statistically significant.

### Data availability

The datasets generated and/or analyzed during the current study are available from the corresponding author upon reasonable request.

## Supplementary Material

Supplementary Table 1

Supplementary Table 2
