# Supplementary material for: Effect of samul-tang on female fertility via RAS signaling pathway in ovaries of aged mice
Source: Aging (Albany NY). 2021 Jun 6;13(11):14829–42. doi: 10.18632/aging.203150 (PMC8221297; doi:10.18632/aging.203150)
Supplement: Supplementary Table 1 [file aging-13-203150-s001.docx]

**Supplementary Table 1. List of genes upregulated in OC+SM mice compared with their expression in OC mice.**

| \| **Gene_Symbol** \| **Gene_ID** \| **OC+SM vs OC.fc** \| **P-value** \| \| --- \| --- \| --- \| --- \| \| **Lgals12** \| lectin, galactose binding, soluble 12 \| 3.730 \| 0.016 \| \| **Ttll10** \| tubulin tyrosine ligase-like family, member 10 \| 3.613 \| 0.028 \| \| **Tmem229a** \| transmembrane protein 229A \| 3.458 \| 0.031 \| \| **Gpr87** \| G protein-coupled receptor 87 \| 3.223 \| 0.022 \| \| **Slc39a2** \| solute carrier family 39 (zinc transporter), member 2 \| 3.121 \| 0.021 \| \| **Mucl1** \| mucin-like 1 \| 2.988 \| 0.017 \| \| **Cyp27b1** \| cytochrome P450, family 27, subfamily b, polypeptide 1 \| 2.909 \| 0.050 \| \| **A530016L24Rik** \| RIKEN cDNA A530016L24 gene \| 2.839 \| 0.025 \| \| **Ocstamp** \| osteoclast stimulatory transmembrane protein \| 2.833 \| 0.007 \| \| **Prss41** \| protease, serine 41 \| 2.799 \| 0.037 \| \| **1700012D14Rik** \| RIKEN cDNA 1700012D14 gene \| 2.785 \| 0.006 \| \| **Spta1** \| spectrin alpha, erythrocytic 1 \| 2.743 \| 0.008 \| \| **Zc3h12d** \| zinc finger CCCH type containing 12D \| 2.676 \| 0.036 \| \| **Ankrd1** \| ankyrin repeat domain 1 (cardiac muscle) \| 2.675 \| 0.028 \| \| **Il1rapl1** \| interleukin 1 receptor accessory protein-like 1 \| 2.650 \| 0.048 \| \| **Gfap** \| glial fibrillary acidic protein \| 2.645 \| 0.024 \| \| **Gm6792** \| predicted gene 6792 \| 2.546 \| 0.018 \| \| **Ggt1** \| gamma-glutamyltransferase 1 \| 2.532 \| 0.023 \| \| **Slc15a2** \| solute carrier family 15 (H+/peptide transporter), member 2 \| 2.493 \| 0.012 \| \| **Serpinb6e** \| serine (or cysteine) peptidase inhibitor, clade B, member 6e \| 2.475 \| 0.041 \| \| **Olah** \| oleoyl-ACP hydrolase \| 2.470 \| 0.018 \| \| **Gm4832** \| predicted gene 4832 \| 2.465 \| 0.007 \| \| **Xrra1** \| X-ray radiation resistance associated 1 \| 2.448 \| 0.001 \| \| **Slitrk5** \| SLIT and NTRK-like family, member 5 \| 2.437 \| 0.036 \| \| **4921511I17Rik** \| RIKEN cDNA 4921511I17 gene \| 2.423 \| 0.027 \| \| **Slfn5os** \| schlafen 5, opposite strand \| 2.423 \| 0.014 \| \| **2210404O09Rik** \| RIKEN cDNA 2210404O09 gene \| 2.411 \| 0.000 \| \| **4833412C05Rik** \| RIKEN cDNA 4833412C05 gene \| 2.404 \| 0.015 \| \| **Lzts1** \| leucine zipper, putative tumor suppressor 1 \| 2.363 \| 0.010 \| \| **Medag** \| mesenteric estrogen dependent adipogenesis \| 2.356 \| 0.010 \| \| **Ddx3y** \| DEAD (Asp-Glu-Ala-Asp) box polypeptide 3, Y-linked \| 2.347 \| 0.003 \| \| **Ptpro** \| protein tyrosine phosphatase, receptor type, O \| 2.329 \| 0.037 \| \| **Tmem117** \| transmembrane protein 117 \| 2.297 \| 0.002 \| \| **Gm16796** \| predicted gene, 16796 \| 2.283 \| 0.037 \| \| **Nwd1** \| NACHT and WD repeat domain containing 1 \| 2.268 \| 0.035 \| \| **1700125H20Rik** \| RIKEN cDNA 1700125H20 gene \| 2.264 \| 0.004 \| \| **AF357359** \| snoRNA AF357359 \| 2.264 \| 0.011 \| \| **Duxbl1** \| double homeobox B-like 1 \| 2.258 \| 0.031 \| \| **1700012C14Rik** \| RIKEN cDNA 1700012C14 gene \| 2.223 \| 0.048 \| \| **Muc19** \| mucin 19 \| 2.218 \| 0.003 \| \| **Gstm3** \| glutathione S-transferase, mu 3 \| 2.192 \| 0.005 \| \| **Gm2694** \| predicted gene 2694 \| 2.171 \| 0.043 \| \| **Gm12596** \| predicted gene 12596 \| 2.158 \| 0.015 \| \| **Klhl32** \| kelch-like 32 \| 2.153 \| 0.032 \| \| **Sprr2a2** \| small proline-rich protein 2A2 \| 2.146 \| 0.022 \| \| **Prtn3** \| proteinase 3 \| 2.126 \| 0.010 \| \| **Rhd** \| Rh blood group, D antigen \| 2.122 \| 0.049 \| \| **Aldob** \| aldolase B, fructose-bisphosphate \| 2.077 \| 0.049 \| \| **Apod** \| apolipoprotein D \| 2.072 \| 0.035 \| \| **Teddm1b** \| transmembrane epididymal protein 1B \| 2.061 \| 0.014 \| \| **Cd55b** \| CD55 molecule, decay accelerating factor for complement B \| 2.055 \| 0.015 \| \| **1700092M07Rik** \| RIKEN cDNA 1700092M07 gene \| 2.048 \| 0.041 \| \| **Tmem215** \| transmembrane protein 215 \| 2.033 \| 0.004 \| \| **4921507L20Rik** \| RIKEN cDNA 4921507L20 gene \| 2.029 \| 0.000 \| \| **Gm20362** \| predicted gene, 20362 \| 2.023 \| 0.014 \| \| **Rbm20** \| RNA binding motif protein 20 \| 2.013 \| 0.000 \| \| **Tnfrsf22** \| tumor necrosis factor receptor superfamily, member 22 \| 2.005 \| 0.023 \| \| **Arhgap8** \| Rho GTPase activating protein 8 \| 1.997 \| 0.029 \| \| **Cutal** \| cutA divalent cation tolerance homolog-like \| 1.994 \| 0.043 \| \| **6430531B16Rik** \| RIKEN cDNA 6430531B16 gene \| 1.993 \| 0.049 \| \| **Gm8709** \| predicted gene 8709 \| 1.990 \| 0.028 \| \| **Fam159a** \| family with sequence similarity 159, member A \| 1.972 \| 0.030 \| \| **Egf** \| epidermal growth factor \| 1.968 \| 0.004 \| \| **Gm5779** \| predicted gene 5779 \| 1.968 \| 0.026 \| \| **Lmntd2** \| lamin tail domain containing 2 \| 1.960 \| 0.027 \| \| **Olr1** \| oxidized low density lipoprotein (lectin-like) receptor 1 \| 1.939 \| 0.047 \| \| **Npy** \| neuropeptide Y \| 1.929 \| 0.031 \| \| **F3** \| coagulation factor III \| 1.904 \| 0.040 \| \| **4933408J17Rik** \| RIKEN cDNA 4933408J17 gene \| 1.902 \| 0.041 \| \| **Cdh13** \| cadherin 13 \| 1.892 \| 0.036 \| \| **Hamp2** \| hepcidin antimicrobial peptide 2 \| 1.885 \| 0.049 \| \| **Rasgrf2** \| RAS protein-specific guanine nucleotide-releasing factor 2 \| 1.841 \| 0.030 \| \| **Slitrk1** \| SLIT and NTRK-like family, member 1 \| 1.840 \| 0.017 \| \| **Zfand4** \| zinc finger, AN1-type domain 4 \| 1.832 \| 0.016 \| \| **Rasgrf1** \| RAS protein-specific guanine nucleotide-releasing factor 1 \| 1.831 \| 0.034 \| \| **Tceal6** \| transcription elongation factor A (SII)-like 6 \| 1.821 \| 0.034 \| \| **Agxt2** \| alanine-glyoxylate aminotransferase 2 \| 1.819 \| 0.049 \| \| **Gcg** \| glucagon \| 1.818 \| 0.035 \| \| **Prdm8** \| PR domain containing 8 \| 1.789 \| 0.044 \| \| **Aqp7** \| aquaporin 7 \| 1.783 \| 0.008 \| \| **Cml5** \| Calmodulin-like protein 5 \| 1.781 \| 0.036 \| \| **Gm8096** \| predicted gene 8096 \| 1.759 \| 0.041 \| \| **4933400C23Rik** \| RIKEN cDNA 4933400C23 gene \| 1.752 \| 0.049 \| \| **Lin28a** \| lin-28 homolog A (C. elegans) \| 1.742 \| 0.037 \| \| **Gng8** \| guanine nucleotide binding protein (G protein), gamma 8 \| 1.734 \| 0.002 \| \| **Elovl2** \| elongation of very long chain fatty acids (FEN1/Elo2, SUR4/Elo3, yeast)-like 2 \| 1.733 \| 0.041 \| \| **Unc5d** \| unc-5 netrin receptor D \| 1.726 \| 0.039 \| \| **Acot4** \| acyl-CoA thioesterase 4 \| 1.706 \| 0.035 \| \| **Snap91** \| synaptosomal-associated protein 91 \| 1.705 \| 0.041 \| \| **Ndrg4** \| N-myc downstream regulated gene 4 \| 1.694 \| 0.014 \| \| **Gpr37** \| G protein-coupled receptor 37 \| 1.687 \| 0.005 \| \| **Nphs2** \| nephrosis 2, podocin \| 1.681 \| 0.011 \| \| **Hist4h4** \| histone cluster 4, H4 \| 1.668 \| 0.036 \| \| **2610035D17Rik** \| RIKEN cDNA 2610035D17 gene \| 1.655 \| 0.014 \| \| **Adh1** \| alcohol dehydrogenase 1 (class I) \| 1.648 \| 0.032 \| \| **Nipa1** \| non imprinted in Prader-Willi/Angelman syndrome 1 homolog (human) \| 1.647 \| 0.047 \| \| **Sectm1b** \| secreted and transmembrane 1B \| 1.633 \| 0.036 \| \| **Lrrc34** \| leucine rich repeat containing 34 \| 1.621 \| 0.017 \| \| **Tmem119** \| transmembrane protein 119 \| 1.616 \| 0.035 \| \| **Mks1** \| Meckel syndrome, type 1 \| 1.615 \| 0.035 \| \| **Ahsg** \| alpha-2-HS-glycoprotein \| 1.614 \| 0.002 \| \| **Cda** \| cytidine deaminase \| 1.598 \| 0.007 \| \| **Ppp1r3d** \| protein phosphatase 1, regulatory subunit 3D \| 1.588 \| 0.002 \| \| **Pdlim3** \| PDZ and LIM domain 3 \| 1.585 \| 0.033 \| \| **Sh3yl1** \| Sh3 domain YSC-like 1 \| 1.577 \| 0.011 \| \| **Fgfr2** \| fibroblast growth factor receptor 2 \| 1.575 \| 0.012 \| \| **Atp6v1c2** \| ATPase, H+ transporting, lysosomal V1 subunit C2 \| 1.564 \| 0.028 \| \| **Ren1** \| renin 1 structural \| 1.553 \| 0.005 \| \| **1700028K03Rik** \| RIKEN cDNA 1700028K03 gene \| 1.548 \| 0.001 \| \| **Raet1e** \| retinoic acid early transcript 1E \| 1.544 \| 0.046 \| \| **Maneal** \| mannosidase, endo-alpha-like \| 1.538 \| 0.019 \| \| **Map6** \| microtubule-associated protein 6 \| 1.536 \| 0.034 \| \| **Zbtb25** \| zinc finger and BTB domain containing 25 \| 1.534 \| 0.000 \| \| **Dnaic2** \| dynein, axonemal, intermediate chain 2 \| 1.521 \| 0.026 \| \| **Hsf4** \| heat shock transcription factor 4 \| 1.513 \| 0.040 \| \| **Perp** \| PERP, TP53 apoptosis effector \| 1.513 \| 0.018 \| \| **Gabra4** \| gamma-aminobutyric acid (GABA) A receptor, subunit alpha 4 \| 1.511 \| 0.017 \| \| **Mtm1** \| X-linked myotubular myopathy gene 1 \| 1.508 \| 0.040 \| \| **Abcg4** \| ATP-binding cassette, sub-family G (WHITE), member 4 \| 1.508 \| 0.005 \| \| **Hoxa7** \| homeobox A7 \| 1.506 \| 0.018 \| \| **Zfp647** \| zinc finger protein 647 \| 1.500 \| 0.023 \| \| **Galnt16** \| UDP-N-acetyl-alpha-D-galactosamine:polypeptide N-acetylgalactosaminyltransferase 16 \| 1.500 \| 0.048 \| |
| --- | --- | --- | --- | --- | --- | --- | --- | --- | --- | --- | --- | --- | --- | --- | --- | --- | --- | --- | --- | --- | --- | --- | --- | --- | --- | --- | --- | --- | --- | --- | --- | --- | --- | --- | --- | --- | --- | --- | --- | --- | --- | --- | --- | --- | --- | --- | --- | --- | --- | --- | --- | --- | --- | --- | --- | --- | --- | --- | --- | --- | --- | --- | --- | --- | --- | --- | --- | --- | --- | --- | --- | --- | --- | --- | --- | --- | --- | --- | --- | --- | --- | --- | --- | --- | --- | --- | --- | --- | --- | --- | --- | --- | --- | --- | --- | --- | --- | --- | --- | --- | --- | --- | --- | --- | --- | --- | --- | --- | --- | --- | --- | --- | --- | --- | --- | --- | --- | --- | --- | --- | --- | --- | --- | --- | --- | --- | --- | --- | --- | --- | --- | --- | --- | --- | --- | --- | --- | --- | --- | --- | --- | --- | --- | --- | --- | --- | --- | --- | --- | --- | --- | --- | --- | --- | --- | --- | --- | --- | --- | --- | --- | --- | --- | --- | --- | --- | --- | --- | --- | --- | --- | --- | --- | --- | --- | --- | --- | --- | --- | --- | --- | --- | --- | --- | --- | --- | --- | --- | --- | --- | --- | --- | --- | --- | --- | --- | --- | --- | --- | --- | --- | --- | --- | --- | --- | --- | --- | --- | --- | --- | --- | --- | --- | --- | --- | --- | --- | --- | --- | --- | --- | --- | --- | --- | --- | --- | --- | --- | --- | --- | --- | --- | --- | --- | --- | --- | --- | --- | --- | --- | --- | --- | --- | --- | --- | --- | --- | --- | --- | --- | --- | --- | --- | --- | --- | --- | --- | --- | --- | --- | --- | --- | --- | --- | --- | --- | --- | --- | --- | --- | --- | --- | --- | --- | --- | --- | --- | --- | --- | --- | --- | --- | --- | --- | --- | --- | --- | --- | --- | --- | --- | --- | --- | --- | --- | --- | --- | --- | --- | --- | --- | --- | --- | --- | --- | --- | --- | --- | --- | --- | --- | --- | --- | --- | --- | --- | --- | --- | --- | --- | --- | --- | --- | --- | --- | --- | --- | --- | --- | --- | --- | --- | --- | --- | --- | --- | --- | --- | --- | --- | --- | --- | --- | --- | --- | --- | --- | --- | --- | --- | --- | --- | --- | --- | --- | --- | --- | --- | --- | --- | --- | --- | --- | --- | --- | --- | --- | --- | --- | --- | --- | --- | --- | --- | --- | --- | --- | --- | --- | --- | --- | --- | --- | --- | --- | --- | --- | --- | --- | --- | --- | --- | --- | --- | --- | --- | --- | --- | --- | --- | --- | --- | --- | --- | --- | --- | --- | --- | --- | --- | --- | --- | --- | --- | --- | --- | --- | --- | --- | --- | --- | --- | --- | --- | --- | --- | --- | --- | --- | --- | --- | --- | --- | --- | --- | --- | --- | --- | --- | --- | --- | --- | --- | --- | --- | --- | --- | --- | --- | --- | --- | --- | --- | --- | --- | --- | --- | --- | --- | --- | --- | --- | --- | --- | --- | --- | --- | --- | --- | --- | --- | --- | --- | --- | --- | --- | --- | --- | --- | --- | --- | --- | --- | --- | --- | --- | --- | --- | --- | --- | --- | --- |

Comparison of the OC and OC+SM mice data revealed 122 differentially upregulated genes with fold changes >1.5, and *P*< 0.05. OC: 40-week-old mice; OC+SM: 40-week-old mice orally administered Samul-tang.
