# Supplementary material for: Effect of samul-tang on female fertility via RAS signaling pathway in ovaries of aged mice
Source: Aging (Albany NY). 2021 Jun 6;13(11):14829–42. doi: 10.18632/aging.203150 (PMC8221297; doi:10.18632/aging.203150)
Supplement: Supplementary Table 2 [file aging-13-203150-s002.docx]

**Supplementary Table 2. List of genes downregulated in OC+SM mice compared with their expression in OC mice.**

| **Gene_Symbol** | **Gene_ID** | **OC+SM vs OC.fc** | **P-value** |
| --- | --- | --- | --- |
| Cyp20a1 | cytochrome P450, family 20, subfamily a, polypeptide 1 | 0.666 | 0.042 |
| Ptprj | protein tyrosine phosphatase, receptor type, J | 0.663 | 0.010 |
| 4930455H04Rik | RIKEN cDNA 4930455H04 gene | 0.663 | 0.026 |
| Ifit3b | interferon-induced protein with tetratricopeptide repeats 3B | 0.661 | 0.020 |
| Mis12 | MIS12 kinetochore complex component | 0.660 | 0.001 |
| Apold1 | apolipoprotein L domain containing 1 | 0.660 | 0.000 |
| Nudcd1 | NudC domain containing 1 | 0.660 | 0.003 |
| Tesc | tescalcin | 0.659 | 0.030 |
| Dhdh | dihydrodiol dehydrogenase (dimeric) | 0.659 | 0.003 |
| BC005537 | cDNA sequence BC005537 | 0.659 | 0.001 |
| Arl5b | ADP-ribosylation factor-like 5B | 0.658 | 0.009 |
| Egr3 | early growth response 3 | 0.657 | 0.050 |
| Gm16523 | predicted gene, 16523 | 0.657 | 0.001 |
| Slc26a2 | solute carrier family 26 (sulfate transporter), member 2 | 0.656 | 0.017 |
| Slfn3 | schlafen 3 | 0.655 | 0.026 |
| Zbtb37 | zinc finger and BTB domain containing 37 | 0.654 | 0.017 |
| Klhl4 | kelch-like 4 | 0.653 | 0.004 |
| Fam189a2 | family with sequence similarity 189, member A2 | 0.652 | 0.018 |
| Iglon5 | IgLON family member 5 | 0.652 | 0.042 |
| Pcdhb14 | protocadherin beta 14 | 0.652 | 0.021 |
| Nf1 | neurofibromatosis 1 | 0.651 | 0.001 |
| Rn45s | ring finger protein 45a | 0.651 | 0.007 |
| Fam81a | family with sequence similarity 81, member A | 0.650 | 0.039 |
| Rassf10 | Ras association (RalGDS/AF-6) domain family (N-terminal) member 10 | 0.650 | 0.041 |
| Gm6297 | predicted gene 6297 | 0.649 | 0.021 |
| Mbd5 | methyl-CpG binding domain protein 5 | 0.649 | 0.006 |
| Srcap | Snf2-related CREBBP activator protein | 0.649 | 0.008 |
| Gm3258 | predicted gene 3258 | 0.644 | 0.040 |
| 5730508B09Rik | RIKEN cDNA 5730508B09 gene | 0.643 | 0.009 |
| Zfhx4 | zinc finger homeodomain 4 | 0.643 | 0.002 |
| Ildr1 | immunoglobulin-like domain containing receptor 1 | 0.643 | 0.045 |
| Narf | nuclear prelamin A recognition factor | 0.643 | 0.002 |
| Arhgap20 | Rho GTPase activating protein 20 | 0.643 | 0.028 |
| Wisp2 | WNT1 inducible signaling pathway protein 2 | 0.641 | 0.040 |
| Apol6 | apolipoprotein L 6 | 0.640 | 0.040 |
| Coro1a | coronin, actin binding protein 1A | 0.639 | 0.003 |
| Dpysl4 | dihydropyrimidinase-like 4 | 0.639 | 0.045 |
| Gm15713 | predicted gene 15713 | 0.635 | 0.044 |
| Zc3h12c | zinc finger CCCH type containing 12C | 0.635 | 0.002 |
| Gm4951 | predicted gene 4951 | 0.635 | 0.033 |
| Lrp8 | low density lipoprotein receptor-related protein 8, apolipoprotein e receptor | 0.635 | 0.006 |
| Pcdh1 | protocadherin 1 | 0.634 | 0.022 |
| Epb4.1l5 | erythrocyte membrane protein band 4.3 like 5 | 0.634 | 0.005 |
| Ccdc171 | coiled-coil domain containing 171 | 0.630 | 0.027 |
| Fnbp1 | formin binding protein 1 | 0.630 | 0.000 |
| Casc1 | cancer susceptibility candidate 1 | 0.630 | 0.017 |
| Cxadr | coxsackie virus and adenovirus receptor | 0.629 | 0.011 |
| Polr3c | polymerase (RNA) III (DNA directed) polypeptide C | 0.627 | 0.020 |
| Tsga10 | testis specific 10 | 0.626 | 0.005 |
| Syne3 | spectrin repeat containing, nuclear envelope family member 3 | 0.625 | 0.011 |
| Il3ra | interleukin 3 receptor, alpha chain | 0.624 | 0.040 |
| Tmem51os1 | Tmem51 opposite strand 1 | 0.624 | 0.014 |
| Mid1 | midline 1 | 0.624 | 0.021 |
| Ppp1r37 | protein phosphatase 1, regulatory subunit 37 | 0.623 | 0.028 |
| Lrrtm2 | leucine rich repeat transmembrane neuronal 2 | 0.620 | 0.003 |
| Trib1 | tribbles pseudokinase 1 | 0.619 | 0.015 |
| Pappa | pregnancy-associated plasma protein A | 0.619 | 0.029 |
| Gm5862 | predicted gene 5862 | 0.619 | 0.025 |
| Nfam1 | Nfat activating molecule with ITAM motif 1 | 0.618 | 0.038 |
| Add2 | adducin 2 (beta) | 0.617 | 0.005 |
| Ribc1 | RIB43A domain with coiled-coils 1 | 0.617 | 0.017 |
| Adap2 | ArfGAP with dual PH domains 2 | 0.616 | 0.028 |
| Isoc1 | isochorismatase domain containing 1 | 0.615 | 0.001 |
| Sorbs2os | sorbin and SH3 domain containing 2, opposite strand | 0.615 | 0.024 |
| Dyrk1b | dual-specificity tyrosine-(Y)-phosphorylation regulated kinase 1b | 0.613 | 0.029 |
| Hist2h2ac | histone cluster 2, H2ac | 0.612 | 0.008 |
| Ltbp1 | latent transforming growth factor beta binding protein 1 | 0.611 | 0.010 |
| Mnda | myeloid cell nuclear differentiation antigen | 0.610 | 0.000 |
| Gipc2 | GIPC PDZ domain containing family, member 2 | 0.609 | 0.022 |
| Runx2 | runt related transcription factor 2 | 0.609 | 0.000 |
| Gk5 | glycerol kinase 5 (putative) | 0.609 | 0.044 |
| Gm1979 | predicted gene 1979 | 0.609 | 0.040 |
| Gm38426 | predicted gene, 38426 | 0.608 | 0.024 |
| Tmem184a | transmembrane protein 184a | 0.606 | 0.029 |
| Nova2 | neuro-oncological ventral antigen 2 | 0.605 | 0.004 |
| Gpc6 | glypican 6 | 0.605 | 0.021 |
| Runx3 | runt related transcription factor 3 | 0.605 | 0.007 |
| Spef2 | sperm flagellar 2 | 0.604 | 0.016 |
| Hist1h4d | histone cluster 1, H4d | 0.604 | 0.046 |
| Ptgfr | prostaglandin F receptor | 0.602 | 0.033 |
| Sox2ot | SOX2 overlapping transcript (non-protein coding) | 0.602 | 0.034 |
| Gm9833 | predicted gene 9833 | 0.601 | 0.007 |
| Sesn2 | sestrin 2 | 0.601 | 0.043 |
| Snord22 | small nucleolar RNA, C/D box 22 | 0.600 | 0.045 |
| Hist1h1e | histone cluster 1, H1e | 0.599 | 0.002 |
| Gm32014 | predicted gene, 32014 | 0.599 | 0.018 |
| Irx5 | Iroquois related homeobox 5 (Drosophila) | 0.598 | 0.043 |
| Slfn1 | schlafen 1 | 0.598 | 0.045 |
| Lrmp | lymphoid-restricted membrane protein | 0.598 | 0.006 |
| A930015D03Rik | RIKEN cDNA A930015D03 gene | 0.597 | 0.011 |
| Slc47a1 | solute carrier family 47, member 1 | 0.597 | 0.006 |
| Slc52a3 | solute carrier protein family 52, member 3 | 0.595 | 0.023 |
| Lipg | lipase, endothelial | 0.595 | 0.021 |
| Scg3 | secretogranin III | 0.594 | 0.048 |
| Gm5766 | predicted gene 5766 | 0.593 | 0.002 |
| Creb3l2 | cAMP responsive element binding protein 3-like 2 | 0.593 | 0.001 |
| 4930467E23Rik | RIKEN cDNA 4930467E23 gene | 0.591 | 0.020 |
| Gm15787 | predicted gene 15787 | 0.588 | 0.026 |
| Ppp1r2-ps3 | protein phosphatase 1, regulatory (inhibitor) subunit 2, pseudogene 3 | 0.588 | 0.010 |
| Il22ra1 | interleukin 22 receptor, alpha 1 | 0.586 | 0.049 |
| Ddx23 | DEAD (Asp-Glu-Ala-Asp) box polypeptide 23 | 0.586 | 0.033 |
| Abca8b | ATP-binding cassette, sub-family A (ABC1), member 8b | 0.585 | 0.020 |
| Fndc9 | fibronectin type III domain containing 9 | 0.585 | 0.031 |
| Nlrx1 | NLR family member X1 | 0.584 | 0.001 |
| Shf | Src homology 2 domain containing F | 0.584 | 0.019 |
| Gpr183 | G protein-coupled receptor 183 | 0.583 | 0.026 |
| Cyyr1 | cysteine and tyrosine-rich protein 1 | 0.579 | 0.005 |
| Snora81 | small nucleolar RNA, H/ACA box 81 | 0.577 | 0.048 |
| Fmod | fibromodulin | 0.576 | 0.005 |
| Odf3l1 | outer dense fiber of sperm tails 3-like 1 | 0.576 | 0.035 |
| Neil1 | nei endonuclease VIII-like 1 (E. coli) | 0.574 | 0.021 |
| 2900009J06Rik | RIKEN cDNA 2900009J06 gene | 0.574 | 0.006 |
| Cracr2a | calcium release activated channel regulator 2A | 0.573 | 0.024 |
| Gm26391 | predicted gene, 26391 | 0.573 | 0.034 |
| Hist1h2bf | histone cluster 1, H2bf | 0.572 | 0.049 |
| Nacad | NAC alpha domain containing | 0.571 | 0.027 |
| Hnrnpul1 | heterogeneous nuclear ribonucleoprotein U-like 1 | 0.571 | 0.013 |
| Zfp57 | zinc finger protein 57 | 0.570 | 0.002 |
| Gm5643 | predicted gene 5643 | 0.570 | 0.000 |
| 1700007J10Rik | RIKEN cDNA 1700007J10 gene | 0.569 | 0.005 |
| Wnk2 | WNK lysine deficient protein kinase 2 | 0.569 | 0.003 |
| Pakap | paralemmin A kinase anchor protein | 0.568 | 0.001 |
| Kirrel | kin of IRRE like (Drosophila) | 0.567 | 0.007 |
| 2900011O08Rik | RIKEN cDNA 2900011O08 gene | 0.564 | 0.046 |
| Gbp9 | guanylate-binding protein 9 | 0.563 | 0.007 |
| Snx9 | sorting nexin 9 | 0.560 | 0.000 |
| Map3k7cl | Map3k7 C-terminal like | 0.560 | 0.027 |
| Gm16386 | predicted gene 16386 | 0.560 | 0.038 |
| Rasal1 | RAS protein activator like 1 (GAP1 like) | 0.559 | 0.018 |
| C130080G10Rik | RIKEN cDNA C130080G10 gene | 0.558 | 0.001 |
| Hist1h1a | histone cluster 1, H1a | 0.554 | 0.029 |
| Rassf8 | Ras association (RalGDS/AF-6) domain family (N-terminal) member 8 | 0.552 | 0.015 |
| Col6a6 | collagen, type VI, alpha 6 | 0.552 | 0.015 |
| Cage1 | cancer antigen 1 | 0.551 | 0.014 |
| Megf9 | multiple EGF-like-domains 9 | 0.549 | 0.025 |
| 4930480K23Rik | RIKEN cDNA 4930480K23 gene | 0.548 | 0.037 |
| Hsd17b7 | hydroxysteroid (17-beta) dehydrogenase 7 | 0.548 | 0.018 |
| Sesn3 | sestrin 3 | 0.547 | 0.002 |
| Snx29 | sorting nexin 29 | 0.547 | 0.043 |
| Itga2 | integrin alpha 2 | 0.546 | 0.001 |
| Prdm10 | PR domain containing 10 | 0.544 | 0.011 |
| Bpifc | BPI fold containing family C | 0.544 | 0.044 |
| Map3k9 | mitogen-activated protein kinase kinase kinase 9 | 0.542 | 0.043 |
| Kcnh4 | potassium voltage-gated channel, subfamily H (eag-related), member 4 | 0.542 | 0.040 |
| Plekha5 | pleckstrin homology domain containing, family A member 5 | 0.542 | 0.000 |
| Fgf13 | fibroblast growth factor 13 | 0.540 | 0.045 |
| Sh3rf3 | SH3 domain containing ring finger 3 | 0.540 | 0.025 |
| Cct6b | chaperonin containing Tcp1, subunit 6b (zeta) | 0.538 | 0.024 |
| Oaz1 | ornithine decarboxylase antizyme 1 | 0.536 | 0.008 |
| Snord49b | small nucleolar RNA, C/D box 49B | 0.535 | 0.001 |
| Dnah8 | dynein, axonemal, heavy chain 8 | 0.534 | 0.017 |
| Gm13289 | predicted gene 13289 | 0.534 | 0.047 |
| 4921533I20Rik | Riken cDNA 4921533I20 gene | 0.534 | 0.039 |
| Hist1h4a | histone cluster 1, H4a | 0.533 | 0.000 |
| Klhl11 | kelch-like 11 | 0.533 | 0.006 |
| Cilp2 | cartilage intermediate layer protein 2 | 0.533 | 0.044 |
| Vwa7 | von Willebrand factor A domain containing 7 | 0.532 | 0.009 |
| Gsg2 | germ cell-specific gene 2 | 0.531 | 0.001 |
| Ybx2 | Y box protein 2 | 0.531 | 0.004 |
| Cfap45 | cilia and flagella associated protein 45 | 0.530 | 0.006 |
| 5031434C07Rik | RIKEN cDNA 5031434C07 gene | 0.530 | 0.026 |
| Gltscr1 | glioma tumor suppressor candidate region gene 1 | 0.530 | 0.012 |
| Hist1h2bq | histone cluster 1, H2bq | 0.526 | 0.012 |
| Nphs1 | nephrosis 1, nephrin | 0.523 | 0.006 |
| Bcas3os1 | breast carcinoma amplified sequence 3, opposite strand 1 | 0.523 | 0.049 |
| Amer2 | APC membrane recruitment 2 | 0.521 | 0.010 |
| Slc7a14 | solute carrier family 7 (cationic amino acid transporter, y+ system), member 14 | 0.518 | 0.006 |
| Xlr5b | X-linked lymphocyte-regulated 5B | 0.517 | 0.022 |
| Gm4759 | predicted gene 4759 | 0.517 | 0.024 |
| Slc2a2 | solute carrier family 2 (facilitated glucose transporter), member 2 | 0.516 | 0.019 |
| Tmem255b | transmembrane protein 255B | 0.513 | 0.043 |
| Gm20735 | predicted gene, 20735 | 0.512 | 0.004 |
| AU040320 | expressed sequence AU040320 | 0.512 | 0.001 |
| Trpa1 | transient receptor potential cation channel, subfamily A, member 1 | 0.511 | 0.046 |
| D130017N08Rik | RIKEN cDNA D130017N08 gene | 0.510 | 0.000 |
| Pclo | piccolo (presynaptic cytomatrix protein) | 0.509 | 0.001 |
| Slfn10-ps | schlafen 10, pseudogene | 0.507 | 0.007 |
| Mgat5 | mannoside acetylglucosaminyltransferase 5 | 0.505 | 0.002 |
| Lpcat1 | lysophosphatidylcholine acyltransferase 1 | 0.505 | 0.036 |
| F10 | coagulation factor X | 0.505 | 0.013 |
| Dgkeos | diacylglycerol kinase, epsilon, opposite strand | 0.502 | 0.021 |
| Gm15350 | predicted gene 15350 | 0.502 | 0.014 |
| Ccdc142 | coiled-coil domain containing 142 | 0.501 | 0.016 |
| Gm20139 | predicted gene, 20139 | 0.501 | 0.049 |
| Xntrpc | Xndc1-transient receptor potential cation channel, subfamily C, member 2 readthrough | 0.499 | 0.002 |
| Gm10509 | predicted gene 10509 | 0.499 | 0.011 |
| Ptar1 | protein prenyltransferase alpha subunit repeat containing 1 | 0.498 | 0.012 |
| Prkag2os1 | protein kinase, AMP-activated, gamma 2 non-catalytic subunit, opposite strand 1 | 0.498 | 0.019 |
| Tbx4 | T-box 4 | 0.498 | 0.008 |
| Acmsd | amino carboxymuconate semialdehyde decarboxylase | 0.497 | 0.030 |
| Nespas | neuroendocrine secretory protein antisense | 0.497 | 0.030 |
| Gan | giant axonal neuropathy | 0.497 | 0.023 |
| Snora24 | small nucleolar RNA, H/ACA box 24 | 0.497 | 0.038 |
| 4930538K18Rik | RIKEN cDNA 4930538K18 gene | 0.490 | 0.024 |
| Nlrc3 | NLR family, CARD domain containing 3 | 0.486 | 0.044 |
| E130218I03Rik | RIKEN cDNA E130218I03 gene | 0.484 | 0.027 |
| Ccdc153 | coiled-coil domain containing 153 | 0.484 | 0.047 |
| Kcnj16 | potassium inwardly-rectifying channel, subfamily J, member 16 | 0.483 | 0.024 |
| Dsc2 | desmocollin 2 | 0.482 | 0.005 |
| Tcp11l1 | t-complex 11 like 1 | 0.481 | 0.005 |
| Far1os | fatty acyl CoA reductase 1, opposite strand | 0.479 | 0.031 |
| 9230117E06Rik | RIKEN cDNA 9230117E06 gene | 0.475 | 0.002 |
| AI661453 | expressed sequence AI661453 | 0.473 | 0.013 |
| Esrrg | estrogen-related receptor gamma | 0.470 | 0.012 |
| Gm16907 | predicted gene, 16907 | 0.468 | 0.029 |
| Ryr1 | ryanodine receptor 1, skeletal muscle | 0.468 | 0.002 |
| Klrk1 | killer cell lectin-like receptor subfamily K, member 1 | 0.466 | 0.035 |
| Zfp369 | zinc finger protein 369 | 0.465 | 0.017 |
| Gm2011 | predicted gene 2011 | 0.465 | 0.019 |
| Ugt3a2 | UDP glycosyltransferases 3 family, polypeptide A2 | 0.465 | 0.044 |
| Gm6592 | predicted gene 6592 | 0.459 | 0.016 |
| Gm4907 | predicted gene 4907 | 0.455 | 0.027 |
| Zfp879 | zinc finger protein 879 | 0.453 | 0.007 |
| Snora75 | small nucleolar RNA, H/ACA box 75 | 0.452 | 0.002 |
| Iqcf5 | IQ motif containing F5 | 0.451 | 0.030 |
| 1700001D01Rik | RIKEN cDNA 1700001D01 gene | 0.450 | 0.035 |
| Ube2j2 | ubiquitin-conjugating enzyme E2J 2 | 0.449 | 0.043 |
| Msi1 | musashi RNA-binding protein 1 | 0.441 | 0.035 |
| Zfp109 | zinc finger protein 109 | 0.440 | 0.003 |
| Fam57a | family with sequence similarity 57, member A | 0.440 | 0.032 |
| Dtx1 | deltex 1, E3 ubiquitin ligase | 0.440 | 0.000 |
| Gm1653 | predicted gene 1653 | 0.434 | 0.047 |
| Erich2 | glutamate rich 2 | 0.433 | 0.017 |
| Ccdc170 | coiled-coil domain containing 170 | 0.432 | 0.017 |
| S1pr5 | sphingosine-1-phosphate receptor 5 | 0.431 | 0.018 |
| Clec4a4 | C-type lectin domain family 4, member a4 | 0.430 | 0.026 |
| Gm5803 | predicted gene 5803 | 0.428 | 0.010 |
| Gm29811 | predicted gene, 29811 | 0.426 | 0.007 |
| Slc26a1 | solute carrier family 26 (sulfate transporter), member 1 | 0.424 | 0.011 |
| Plcd4 | phospholipase C, delta 4 | 0.424 | 0.007 |
| Prima1 | proline rich membrane anchor 1 | 0.423 | 0.021 |
| Ptprt | protein tyrosine phosphatase, receptor type, T | 0.422 | 0.007 |
| Sgol2b | shugoshin like 2b (S. pombe) | 0.422 | 0.037 |
| Olfr33 | olfactory receptor 33 | 0.420 | 0.041 |
| 4930447A16Rik | RIKEN cDNA 4930447A16 gene | 0.418 | 0.046 |
| BB031773 | expressed sequence BB031773 | 0.413 | 0.003 |
| Pnma1 | paraneoplastic antigen MA1 | 0.413 | 0.041 |
| Tnni1 | troponin I, skeletal, slow 1 | 0.409 | 0.001 |
| Cpb1 | carboxypeptidase B1 (tissue) | 0.408 | 0.031 |
| Gucy1b2 | guanylate cyclase 1, soluble, beta 2 | 0.404 | 0.021 |
| Nsun3 | NOL1/NOP2/Sun domain family member 3 | 0.403 | 0.003 |
| Ttc36 | tetratricopeptide repeat domain 36 | 0.403 | 0.000 |
| Cacna1f | calcium channel, voltage-dependent, alpha 1F subunit | 0.400 | 0.004 |
| Tctex1d1 | Tctex1 domain containing 1 | 0.398 | 0.004 |
| Gm12238 | predicted gene 12238 | 0.394 | 0.006 |
| Gm15941 | predicted gene 15941 | 0.389 | 0.042 |
| 2010001E11Rik | RIKEN cDNA 201001E11 gene | 0.388 | 0.042 |
| 1700054K19Rik | RIKEN cDNA 1700054K19 gene | 0.381 | 0.031 |
| C130060C02Rik | RIKEN cDNA C130060C02 gene | 0.380 | 0.021 |
| Rimbp3 | RIMS binding protein 3 | 0.379 | 0.020 |
| Snord89 | small nucleolar RNA, C/D box 89 | 0.379 | 0.003 |
| Il1rl2 | interleukin 1 receptor-like 2 | 0.373 | 0.001 |
| 9930111J21Rik1 | RIKEN cDNA 9930111J21 gene 1 | 0.369 | 0.036 |
| AA619741 | expressed sequence AA619741 | 0.364 | 0.009 |
| Cyp2a22 | cytochrome P450, family 2, subfamily a, polypeptide 22 | 0.360 | 0.012 |
| Cfap44 | cilia and flagella associated protein 44 | 0.351 | 0.031 |
| Vmn2r8 | vomeronasal 2, receptor 8 | 0.345 | 0.000 |
| Mixl1 | Mix1 homeobox-like 1 (Xenopus laevis) | 0.334 | 0.037 |
| Tmc5 | transmembrane channel-like gene family 5 | 0.331 | 0.010 |
| Pcdhga10 | protocadherin gamma subfamily A, 10 | 0.327 | 0.008 |
| Klhdc1 | kelch domain containing 1 | 0.326 | 0.017 |
| Dusp15 | dual specificity phosphatase-like 15 | 0.320 | 0.025 |
| Hus1b | HUS1 checkpoint clamp component B | 0.317 | 0.031 |
| Gm15326 | predicted gene 15326 | 0.317 | 0.028 |
| Cdh6 | cadherin 6 | 0.312 | 0.033 |
| Fam92b | family with sequence similarity 92, member B | 0.310 | 0.013 |
| Shc3 | src homology 2 domain-containing transforming protein C3 | 0.302 | 0.006 |
| Akap6 | A kinase (PRKA) anchor protein 6 | 0.279 | 0.023 |
| Rbm11 | RNA binding motif protein 11 | 0.245 | 0.007 |
| Rnf138rt1 | ring finger protein 138, retrogene 1 | 0.237 | 0.003 |
| Hhipl2 | hedgehog interacting protein-like 2 | 0.181 | 0.013 |

Comparison of the OC and OC+SM mice data revealed 272 differentially downregulated genes with fold changes >1.5, and *P*< 0.05. OC: 40-week-old mice; OC+SM: 40-week-old mice orally administered Samul-tang.
